# Scutellarein nanoparticles suppress colon cancer via PHB2-mediated mitophagy inhibition with modulatory effects on tumor-associated depression and osteotropic migration

**DOI:** 10.3389/fpubh.2026.1996414

**Published:** 2026-10-28

**Authors:** Yuanzhi Li, Hao Xu, Zonglin Cai, Shimei Liu, Xuqiang Luo, Jinghua Liang, Yingying Liao

**Affiliations:** Department of Anorectal Surgery, Shenzhen TCM Anorectal Hospital (Futian), Shenzhen, Guangdong, China

**Keywords:** colon cancer, mitophagy, PHB2, scutellarein nanoparticles, tumor-associated depression, osteotropic migration

## Abstract

**Background:**

Colorectal cancer progression involves mitophagy, in which Prohibitin 2 (PHB2) regulates the PARL–PGAM5–PINK1 axis. Scutellarein has antitumor activity but poor bioavailability. We developed integrin αvβ3-targeted β-cyclodextrin-based scutellarein nanoparticles (Scu-NPs), which enhance the solubility, stability, and tumor-targeting of scutellarein, and investigated whether they suppress colon cancer via PHB2-mediated mitophagy inhibition and modulate tumor-associated depression and osteotropic migration.

**Methods:**

PHB2 expression and mitochondrial localization were examined in colon cancer cells. SW480 cells with PHB2 overexpression were treated with Scu or Scu-NPs. Proliferation, migration, invasion, apoptosis, mitochondrial function, and mitophagy-related proteins were assessed. Subcutaneous xenografts, a chronic unpredictable mild stress tumor–depression model, behavioral tests, osteotropic migration assays, and bioinformatics were used.

**Results:**

PHB2 was highly expressed and mitochondria-localized in colon cancer cells. Scu-NPs downregulated PHB2, increased PARL, decreased PGAM5, PINK1, Parkin, and LC3-II/LC3-I, and increased P62, indicating mitophagy inhibition. Scu-NPs induced mitochondrial dysfunction, ROS accumulation, apoptosis, and inhibited proliferation, migration, and invasion; PHB2 overexpression partially reversed these effects. *In vivo*, Scu-NPs suppressed xenograft growth and improved depressive-like behaviors, with PHB2 overexpression attenuating these effects. *In vitro*, Scu-NPs reduced colon cancer cell migration toward osteoblast-conditioned medium, whereas PHB2 overexpression partially reversed this effect.

**Conclusion:**

Scu-NPs suppress colon cancer by downregulating PHB2 and inhibiting mitophagy. PHB2 may serve as a shared molecular node linking tumor progression, depressive-like behaviors, and osteotropic migration, supporting Scu-NPs as a multi-target strategy.

## Introduction

Colorectal cancer is one of the most common malignancies worldwide, with high morbidity and mortality (1). Despite advances in surgery, radiotherapy, chemotherapy, and targeted therapy, patients still face high recurrence, metastasis, chemotherapy resistance, and significant toxicity (2). Thus, identifying novel molecular targets and safer therapeutic agents remains clinically important. Recent studies have revealed that mitophagy plays a critical role in tumor progression (3, 4). Tumor cells enhance mitophagy to eliminate damaged mitochondria and maintain quality control, thereby adapting to metabolic stress, evading apoptosis, and promoting survival and drug resistance. Targeted inhibition of mitophagy is therefore considered a promising anticancer strategy (5).

Among mitophagy regulatory networks, the PTEN-induced kinase 1 (PINK1)/Parkin pathway is one of the best-characterized mechanisms (6, 7). Upon loss of mitochondrial membrane potential, PINK1 stabilizes on the outer membrane and recruits Parkin, which ubiquitinates outer membrane proteins and links to LC3 via adaptors to mediate mitophagy (8). PHB2, an inner mitochondrial membrane mitophagy receptor, binds microtubule-associated protein 1 light chain 3 (LC3)-II through its LIR motif and interacts with the inner membrane protease presenilin-associated rhomboid-like protein (PARL) (9, 10). PHB2 depletion activates PARL, which cleaves phosphoglycerate mutase family member 5 (PGAM5) and PINK1, preventing PINK1 stabilization and suppressing mitophagy (11). Thus, the PHB2–PARL–PGAM5–PINK1 axis is a critical mitophagy-regulating cascade. PHB2 is aberrantly overexpressed in colon cancer and may serve as a biomarker, suggesting that targeting this axis could inhibit mitophagy and exert antitumor effects (12, 13).

Scutellarein, a major flavonoid from Scutellaria barbata, exhibits potent antitumor activity (14). Our previous studies showed that scutellarein inhibits colon cancer cell proliferation, migration, and invasion, and induces apoptosis via metastasis-associated in colon cancer 1 (MACC1) downregulation and CDC4-mediated RAGE ubiquitination, with selective cytotoxicity and low toxicity (15, 16). However, its clinical application is limited by poor solubility, rapid metabolism, and low bioavailability (17). Nanocarriers can improve solubility, stability, targeting, and circulation time (18, 19). We previously constructed integrin αvβ3-targeted β-cyclodextrin-based scutellarein nanoparticles (Scu-NPs) with favorable biocompatibility and targeting, which significantly enhanced the antiproliferative effect of scutellarein, potentially through PHB2 downregulation (20).

Colorectal cancer patients face a significant comorbidity burden (21). Approximately 20–40% develop depressive symptoms, which may promote tumor progression via neuroendocrine–immune–inflammatory pathways (22, 23). Bone metastasis is a common complication in advanced disease, causing pain, fractures, and reduced survival (24). Emerging evidence links mitochondrial dysfunction not only to tumorigenesis but also to depression and bone metabolic disorders, positioning mitophagy pathways as a potential molecular bridge connecting these conditions (25–27). Our preliminary bioinformatics analysis suggested PHB2 as a potential node in cancer–depression comorbidity, and both animal and *in vitro* migration assays observed modulatory trends of Scu-NPs on depressive-like behaviors and osteotropic migration, though the mechanisms remain unclear.

Based on these observations, this study investigated whether Scu-NPs, which enhance the solubility, stability, and tumor-targeting of scutellarein, exert anti-colon cancer effects by downregulating PHB2, modulating the PARL–PGAM5–PINK1 axis, and suppressing mitophagy. Using cell lines and a xenograft model, we examined the effects of Scu-NPs on this axis and mitophagy, and analyzed their impact on proliferation, apoptosis, and migration. Additionally, using bioinformatics, a tumor–depression comorbidity mouse model, and osteotropic migration assays, we preliminarily explored the modulatory effects of Scu-NPs on depressive-like behaviors and osteotropic migration, aiming to provide initial support for PHB2 as a shared molecular node connecting tumor growth, depressive-like behaviors, and osteotropic migration.

## Materials and methods

### Cell culture

Human colon cancer cell lines SW480, HCT-116, HT-29, and human normal colon epithelial cells HCoEpiC were cultured in DMEM supplemented with 10% fetal bovine serum and 1% penicillin–streptomycin at 37 °C in a 5% CO₂ incubator. Cell recovery and subculture were performed according to standard procedures. SW480 cells were selected for subsequent experiments because they exhibited the highest PHB2 expression among the three colon cancer cell lines tested.

### Cell transfection

#### Transient transfection

Cells were seeded in 6-well plates at 2 × 10^5^ cells/well. The following day, 2.5 μg of plasmid (human PHB2 full-length CDS, GenBank accession No. NM_001144831, cloned into pcDNA3.1(+) vector; empty vector pcDNA3.1(+) as control) was transfected into cells using Lipo8000. Cells were harvested for experiments 24–48 h post-transfection.

#### Stable transfection

SW480 cells were transfected with the above plasmid, and 48 h later, cells were selected with 800 μg/mL G418 for 2 weeks to establish stable PHB2-overexpressing cell lines, which were validated by Western blot and qPCR before use.

### Cell functional assays

#### CCK-8

Cells were seeded in 96-well plates at 3,000 cells/well. After 24 h, CCK-8 reagent was added and incubated for 2 h, followed by absorbance measurement at 450 nm.

#### Colony formation

Cells were seeded in 6-well plates at 1,000 cells/well and cultured for 14 days. Colonies were fixed, stained with crystal violet, and counted.

#### Wound healing assay

Cells were grown to confluence, scratched with a pipette tip, and cultured in serum-free medium for 24 h. Wound closure area was measured.

#### Transwell migration assay

Cells (1 × 10^5^) in serum-free medium were seeded in the upper chamber; the lower chamber contained medium with 10% FBS. After 24 h, migrated cells were counted. For invasion assays, the upper chamber was pre-coated with Matrigel.

#### Osteotropic migration assay

The lower chamber contained osteoblast-conditioned medium (prepared from MC3T3-E1 cell culture supernatant); the remaining steps were the same as the migration assay. Experimental groups included Control, Scu, Scu-NPs, OverExp-NC, OverExp-PHB2, and Scu-NPs + OverExp-PHB2.

### Apoptosis and mitochondrial function assays

#### Apoptosis

Cells were collected and stained with Annexin V-APC/PI, and apoptosis rate was measured by flow cytometry.

#### Mitochondrial membrane potential (ΔΨm)

Cells were stained with JC-1 at 37 °C for 20 min, and red/green fluorescence was observed under confocal microscopy.

#### ROS detection

Cells were incubated with DCFH-DA probe for 20 min, and fluorescence intensity was measured using a microplate reader.

#### ATP detection

ATP levels were determined by chemiluminescence according to the manufacturer’s instructions.

### Immunofluorescence, qPCR, and Western blot

#### Immunofluorescence

Cells were fixed, permeabilized, and blocked, then incubated with primary antibodies against PHB2 and Grp75 at 4 °C overnight, followed by fluorescent secondary antibodies at room temperature for 1 h. Nuclei were counterstained with DAPI, and images were acquired by confocal microscopy.

#### qPCR

Total RNA was extracted using RNAiso Plus and reverse-transcribed to cDNA. qPCR was performed using SYBR Green with GAPDH as internal control. Relative expression was calculated using the 2^−^ΔΔCt method. The primer sequences used for qPCR are listed in Table 1.

**Table 1:** Primer sequence information.

Primer	Sequence(5′-3′)	Product(bp)
PHB2-F	GCTGGACTACGAGGAACGAG	164
PHB2-R	CCAGGATGAGGCTGAAGTCC	
hGAPDH-F1	TTCGTCATGGGTGTGAACCA	170
hGAPDH-R1	GTCTTCTGGGTGGCAGTGAT	

#### Western blot

Cells or tissues were lysed, and protein concentration was determined by BCA assay. Proteins were separated by SDS-PAGE, transferred to membranes, blocked with 5% non-fat milk, incubated with primary antibodies at 4 °C overnight, followed by HRP-conjugated secondary antibodies at room temperature for 1 h. Signals were visualized by ECL. β-actin served as loading control.

### Subcutaneous xenograft tumor model in nude mice

Male BALB/c nude mice (6–8 weeks old) were subcutaneously injected in the right axilla with 5 × 10^6^ SW480 cells mixed with Matrigel (1:1). Mice were divided into four groups (n = 6 per group): Control, Scu, Scu-NPs, and Scu-NPs + OverExp-PHB2. The Control, Scu, and Scu-NPs groups received SW480 cells transfected with empty vector, while the Scu-NPs + OverExp-PHB2 group received SW480 cells stably overexpressing PHB2. When tumor volume reached approximately 100 mm^3^, treatment was initiated: Control group received saline via tail vein; Scu group received free scutellarein at 20 mg/kg; Scu-NPs group received scutellarein nanoparticles at 20 mg/kg; and Scu-NPs + OverExp-PHB2 group received the same dose of scutellarein nanoparticles. Drugs were administered every 2 days for 3 consecutive weeks. Tumor length and width were measured every 2 days, and tumor volume was calculated as V = (L × W^2^)/2. At the end of treatment, mice were euthanized, tumors were excised and weighed, and tissues were processed for HE staining, immunohistochemistry, and Western blot.

### Tumor–depression comorbidity mouse model and behavioral tests

Male BALB/c nude mice (6–8 weeks old) were subcutaneously injected in the right axilla with 5 × 10^6^ SW480 cells mixed with Matrigel (1:1). Mice were randomly assigned to four groups (*n* = 6 per group) using a random number table: normal, model, Scu-NPs, and Scu-NPs + PHB2-OE. The model group and Scu-NPs group received SW480 cells transfected with empty vector, while the Scu-NPs + PHB2-OE group received SW480 cells stably overexpressing PHB2. The normal group received neither tumor inoculation nor stress exposure. Chronic unpredictable mild stress (CUMS) was initiated on the day of tumor inoculation and included food/water deprivation, cage tilting, damp bedding, overnight illumination, restraint, and cold-water swimming, with 1–2 stressors randomly applied daily for 4 weeks. Drug administration commenced simultaneously with CUMS: Scu-NPs and Scu-NPs + PHB2-OE groups received scutellarein nanoparticles at 20 mg/kg via tail vein every 2 days for 4 weeks (total of 14 doses); normal and model groups received equivalent volumes of saline. Behavioral tests were performed 24 h after the last administration.

#### Tail suspension test

Mice were suspended by the tail for 6 min, and immobility time was recorded during the last 4 min.

#### Forced swimming test

Mice were placed in a cylinder (15 cm water depth) for 6 min, and immobility time was recorded during the last 4 min.

#### Sucrose preference test

After training, mice were given free access to 1% sucrose solution and normal drinking water for 24 h, and sucrose preference rate was calculated.

### Histological analysis

#### HE staining

Paraffin sections were deparaffinized, rehydrated, stained with hematoxylin and eosin, and observed under light microscopy.

#### Immunohistochemistry

Paraffin sections (4 μm) were deparaffinized, rehydrated, and subjected to antigen retrieval. After blocking endogenous peroxidase with 3% H₂O₂ and blocking with 5% BSA, sections were incubated with primary antibodies against COX4, PINK1, PHB2, Bax, and Bcl-2 at 4 °C overnight, followed by HRP-conjugated secondary antibody. Immunoreactivity was visualized with DAB, and nuclei were counterstained with hematoxylin. Images were captured under a light microscope, and positive staining was quantified using ImageJ.

### Bioinformatics analysis

Transcriptomic data for colon adenocarcinoma (GSE44076) and depression (GSE98793) were obtained from the GEO database. Differential expression analysis was performed using the limma package. Intersecting differentially expressed genes between the two diseases were subjected to GO and KEGG enrichment analysis via Enrichr. The expression pattern of PHB2 in colon cancer was validated using TCGA-COAD and GTEx data from UCSC Xena.

### Statistical analysis

Data are presented as mean ± SD. Multiple group comparisons were performed using one-way ANOVA followed by Tukey’s *post hoc* test. Tumor volume and CCK-8 data at multiple time points were analyzed by two-way repeated-measures ANOVA followed by Tukey’s *post hoc* test. Behavioral data were analyzed by one-way ANOVA followed by Tukey’s *post hoc* test. All analyses were performed using GraphPad Prism. *p* < 0.05 was considered statistically significant. *p < 0.05, **p < 0.01, ***p < 0.001, ****p < 0.0001.

## Results

### Expression and mitochondrial localization of PHB2 in colon cancer cells

To clarify the potential role of PHB2 in colon cancer, we first examined PHB2 expression in normal colon epithelial cells HCoEpiC and colon cancer cell lines SW480, HCT-116, and HT-29. Western blot and RT-qPCR results demonstrated that PHB2 protein and total mRNA levels were significantly elevated in all three colon cancer cell lines compared to HCoEpiC (*p* < 0.0001), with the highest expression in SW480 cells, followed by HCT-116 and HT-29. Immunofluorescence revealed clear co-localization of PHB2 with the mitochondrial marker Grp75 in both HCoEpiC and colon cancer cells (Figure 1). These results indicate that PHB2 is highly expressed and predominantly localized to mitochondria in colon cancer cells. Given the highest expression level in SW480 cells, this cell line was selected for subsequent experiments.

**Figure 1:**
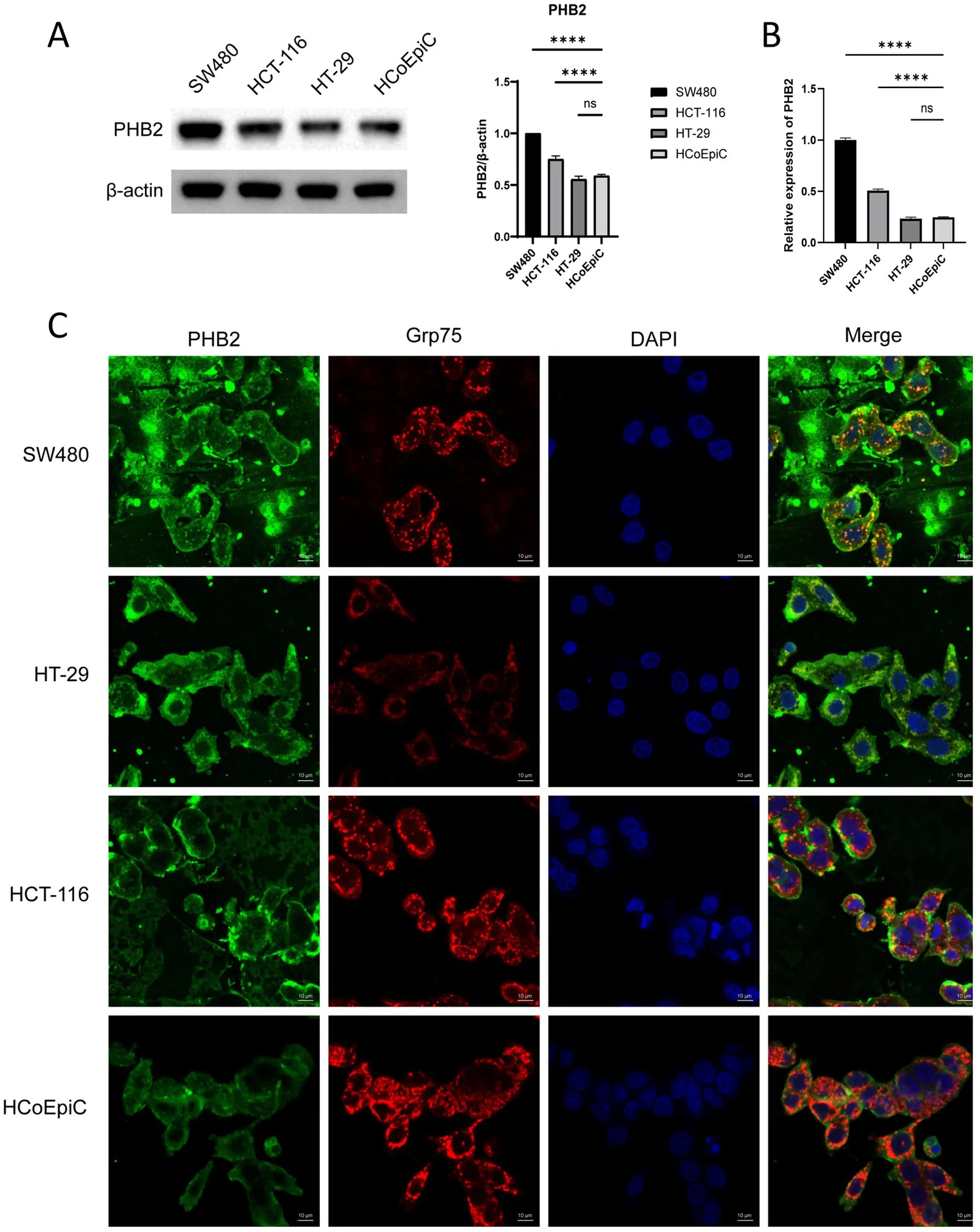
PHB2 is highly expressed and localizes to mitochondria in colon cancer cells. **(A)** Western blot analysis of PHB2 protein expression in normal colon epithelial cells (HCoEpiC) and colon cancer cell lines (SW480, HCT-116, HT-29). The bar graph shows quantitative analysis. **(B)** RT-qPCR analysis of PHB2 mRNA levels in total RNA from the indicated cells. GAPDH served as the internal control, and relative PHB2 expression was normalized to HCoEpiC cells. **(C)** Representative immunofluorescence images showing co-localization of PHB2 (green) with the mitochondrial marker Grp75 (red) in HCoEpiC and colon cancer cells. Nuclei were counterstained with DAPI (blue). Data are presented as mean ± SD. ****p < 0.0001 vs. HCoEpiC. PHB2 expression was highest in SW480 cells, which were therefore selected for subsequent experiments.

### Scu-NPs downregulate PHB2 and suppress mitophagy via the PHB2–PARL–PGAM5–PINK1 axis

Transmission electron microscopy revealed normal short rod-shaped mitochondria in the Control group; mild mitochondrial swelling in the Scu group; severe swelling, cristae disruption, and even fragmentation in the Scu-NPs group; elongated tubular mitochondria with intact morphology in the OverExp-PHB2 group; and partial reversal of Scu-NPs-induced mitochondrial damage in the Scu-NPs + OverExp-PHB2 group compared with the Scu-NPs group, although mitochondrial morphology remained less intact than in the OverExp-PHB2 group. Immunofluorescence showed reduced PHB2 fluorescence intensity after Scu-NPs treatment, while PHB2 overexpression increased fluorescence; Scu-NPs partially reversed the PHB2 elevation induced by overexpression. Quantitative Western blot analysis revealed that, compared with the Control group, the Scu-NPs group exhibited significant reductions in PHB2, PGAM5, PINK1, and Parkin protein levels (*p* < 0.0001), decreased LC3-II/LC3-I ratio (*p* < 0.001), and increased P62 and PARL levels (*p* < 0.001). The Scu group showed similar trends but with lesser magnitude. Compared with the OverExp-NC group, the OverExp-PHB2 group showed opposite changes in the above proteins. Moreover, compared with the OverExp-PHB2 group, the Scu-NPs + OverExp-PHB2 group showed significantly decreased PHB2, PGAM5, PINK1, Parkin, and LC3-II/LC3-I ratio, with increased P62 and PARL (Figure 2). Collectively, these results suggest that Scu-NPs downregulate PHB2 and relieve its inhibitory effect on PARL, accompanied by reduced PGAM5 and PINK1 protein levels and suppression of PHB2-related mitophagy signaling. This protein expression pattern is consistent with the known regulatory model in which PARL is activated upon PHB2 depletion, although direct detection of PARL cleavage products requires further verification.

**Figure 2:**
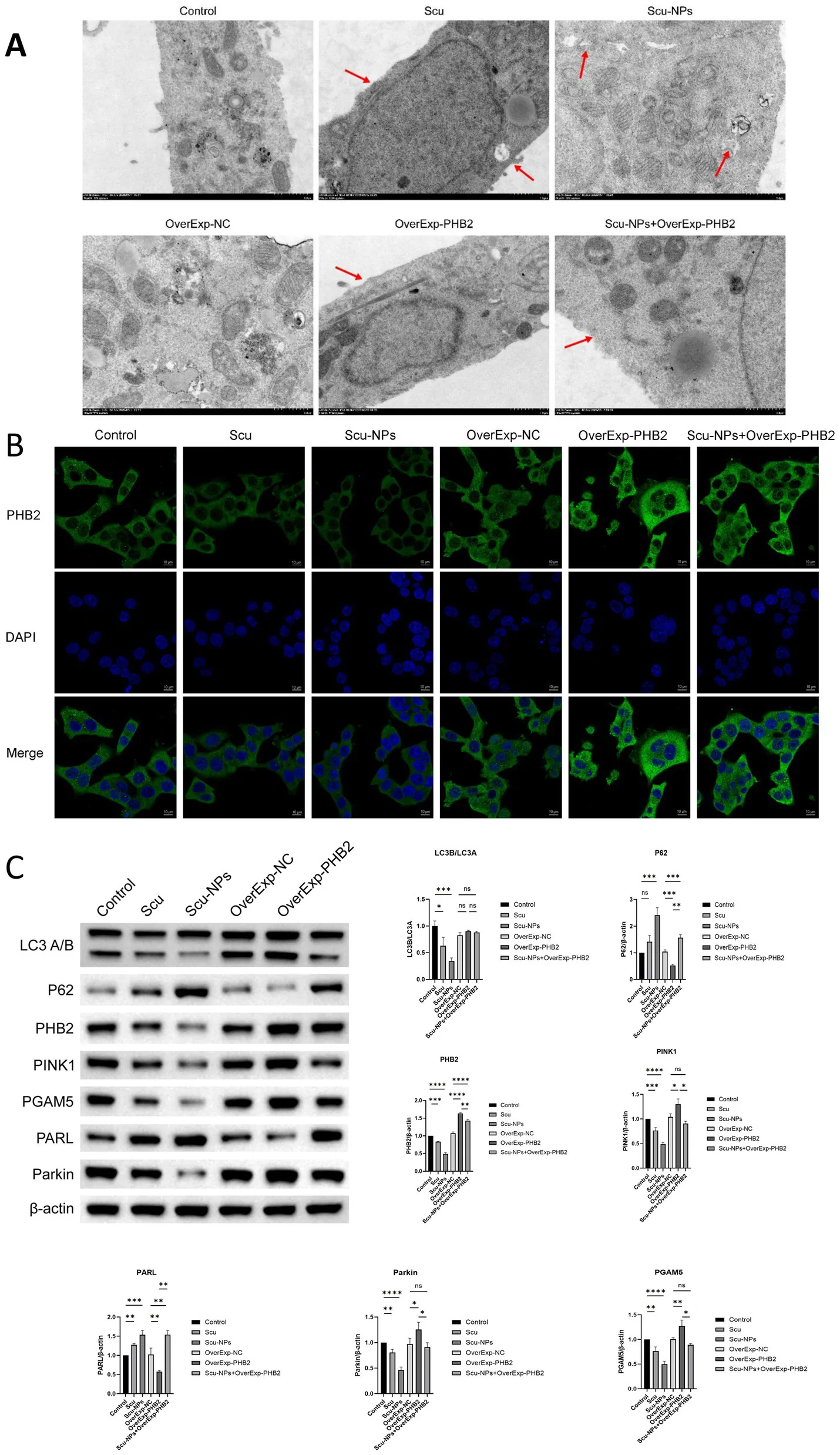
Scu-NPs downregulate PHB2 and inhibit mitophagy via the PHB2–PARL–PGAM5–PINK1 axis. **(A)** Transmission electron microscopy (TEM) images showing mitochondrial morphology and autophagosome formation in the indicated groups. **(B)** Representative immunofluorescence images of PHB2 expression and subcellular distribution. Fluorescence intensity was not quantitatively analyzed. **(C)** Western blot analysis and quantitative analysis of LC3-II/LC3-I, P62, PHB2, PARL, PGAMs, PINK1, and Parkin in the indicated groups (Control, Scu, Scu-NPs, OverExp-NC, OverExp-PHB2, and Scu-NPs + OverExp-PHB2). Data are presented as mean ± SD. *p < 0.05, **p < 0.01, ***p < 0.001, ****p < 0.0001.

### Scu-NPs induce mitochondrial dysfunction

Mitochondrial function assays showed that, compared with the Control group, the Scu-NPs group exhibited significantly decreased JC-1 red/green fluorescence ratio (*p* < 0.0001), markedly reduced ATP content (*p* < 0.0001), and elevated ROS levels (*p* < 0.0001); the Scu group showed weaker changes in these parameters. Compared with OverExp-NC group, the OverExp-PHB2 group showed increased JC-1 ratio and ATP content, with reduced ROS levels. Importantly, compared with OverExp-PHB2 group, the Scu-NPs + OverExp-PHB2 group displayed significantly decreased JC-1 ratio and ATP content, and increased ROS levels (Figure 3). These results indicate that Scu-NPs cause loss of mitochondrial membrane potential, energy metabolism impairment, and oxidative stress elevation, while PHB2 overexpression partially rescues mitochondrial function, suggesting that PHB2 downregulation is a key contributor to Scu-NPs-induced mitochondrial dysfunction.

**Figure 3:**
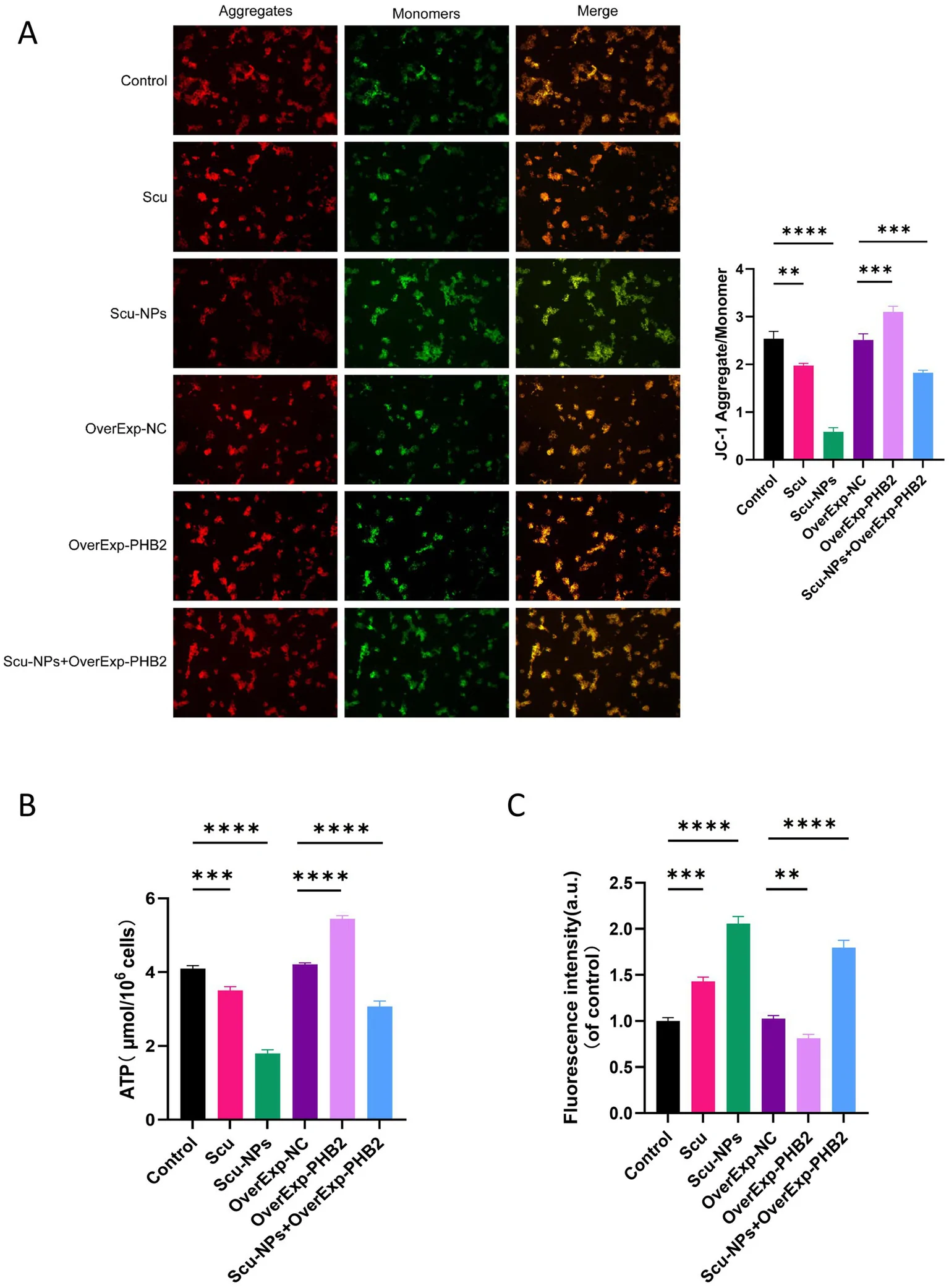
Scu-NPs induce mitochondrial dysfunction in colon cancer cells. **(A)** JC-1 staining for mitochondrial membrane potential (red/green fluorescence ratio). **(B)** ATP content measured by chemiluminescence. **(C)** ROS levels measured by DCFH-DA fluorescence. Groups: Control, Scu, Scu-NPs, OverExp-NC, OverExp-PHB2, and Scu-NPs + OverExp-PHB2. Data are presented as mean ± SD. *p < 0.05, **p < 0.01, ***p < 0.001, ****p < 0.0001.

### Scu-NPs inhibit proliferation, migration, and invasion of colon cancer cells

Colony formation and CCK-8 assays revealed that, compared with the Control group, cell proliferation was mildly inhibited in the Scu group and most markedly inhibited in the Scu-NPs group. PHB2 overexpression promoted cell proliferation, whereas Scu-NPs treatment significantly suppressed this effect. Wound healing and Transwell invasion assays demonstrated that Scu-NPs significantly inhibited migration and invasion of colon cancer cells, with greater potency than free Scu. Compared with the OverExp-PHB2 group, the Scu-NPs + OverExp-PHB2 group showed markedly reduced migration and invasion capacities (Figure 4). These results indicate that Scu-NPs effectively inhibit colon cancer cell proliferation, migration, and invasion *in vitro*, and this effect is at least partially dependent on PHB2 downregulation.

**Figure 4:**
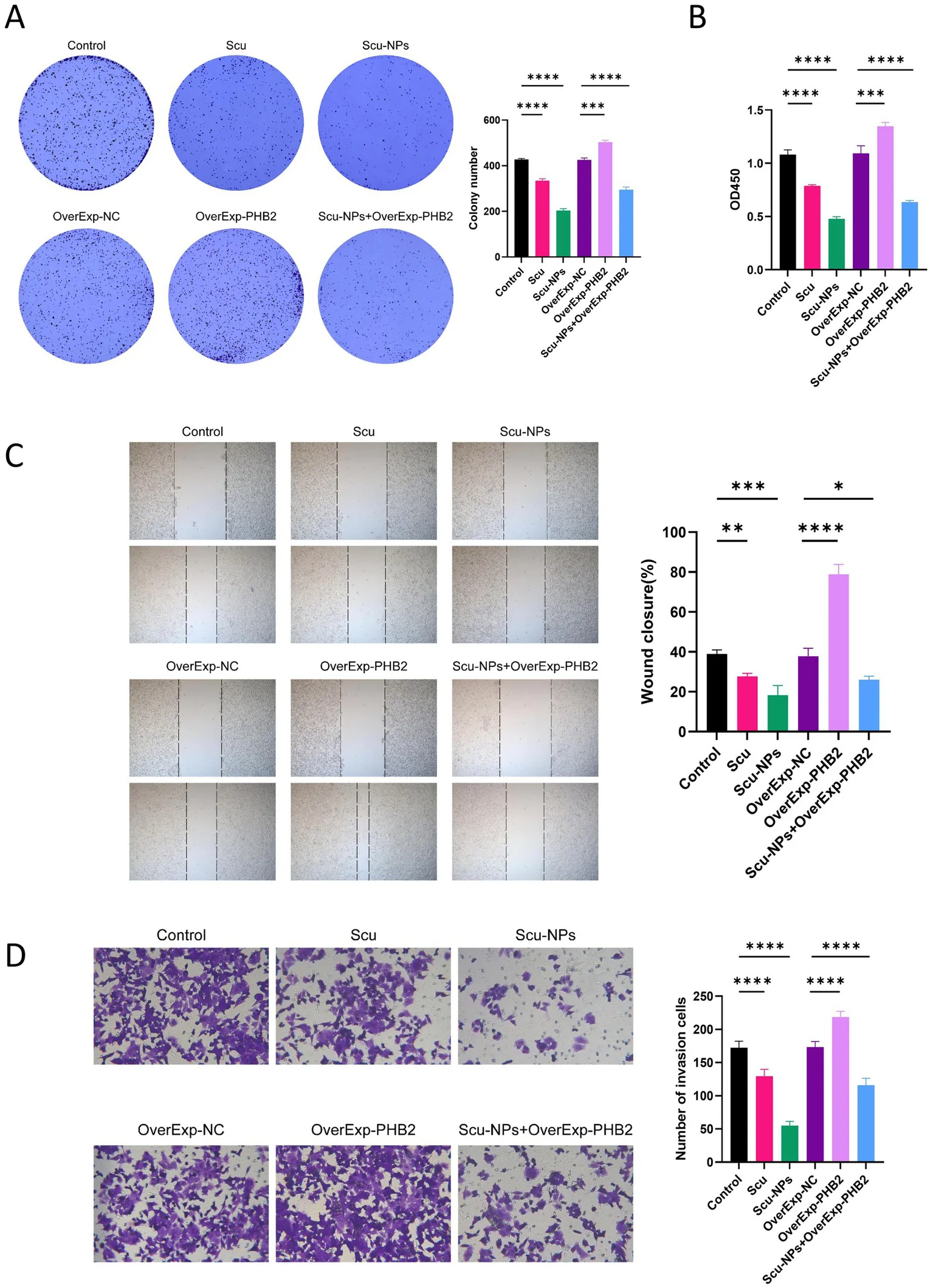
Scu-NPs inhibit proliferation, migration, and invasion of colon cancer cells. **(A)** Colony formation assay showing long-term proliferative capacity. **(B)** CCK-8 assay showing cell viability. **(C)** Wound healing assay showing cell migration. **(D)** Transwell invasion assay. Groups: Control, Scu, Scu-NPs, OverExp-NC, OverExp-PHB2, and Scu-NPs + OverExp-PHB2. Data are presented as mean ± SD. *p < 0.05, **p < 0.01, ***p < 0.001, ****p < 0.0001.

### Scu-NPs induce apoptosis and modulate expression of proliferation-, migration-, and apoptosis-related proteins in colon cancer cells

Flow cytometry results showed that the apoptosis rate was 9.92 ± 0.15% in the Control group, increased to 15.98 ± 0.67% in the Scu group, and further increased to 25.14 ± 0.51% in the Scu-NPs group. The apoptosis rate in the OverExp-PHB2 group was lower than that in the OverExp-NC group, while the Scu-NPs + OverExp-PHB2 group (20.22 ± 0.25%) exhibited a significantly higher apoptosis rate compared with the OverExp-PHB2 group (5.08 ± 0.24%). qPCR and Western blot analyses showed that Scu-NPs significantly downregulated MMP-2, MMP-9, Ki-67, and Bcl-2 expression, and upregulated Bax and Caspase-3 expression; the Scu group showed weaker effects. PHB2 overexpression upregulated MMP-2, MMP-9, Ki-67, and Bcl-2 while downregulating Bax and Caspase-3, and these changes were partially reversed by Scu-NPs treatment (Figure 5). These results suggest that Scu-NPs suppress colon cancer cell proliferation- and migration-related molecular expression and activate apoptotic pathways through downregulation of PHB2.

**Figure 5:**
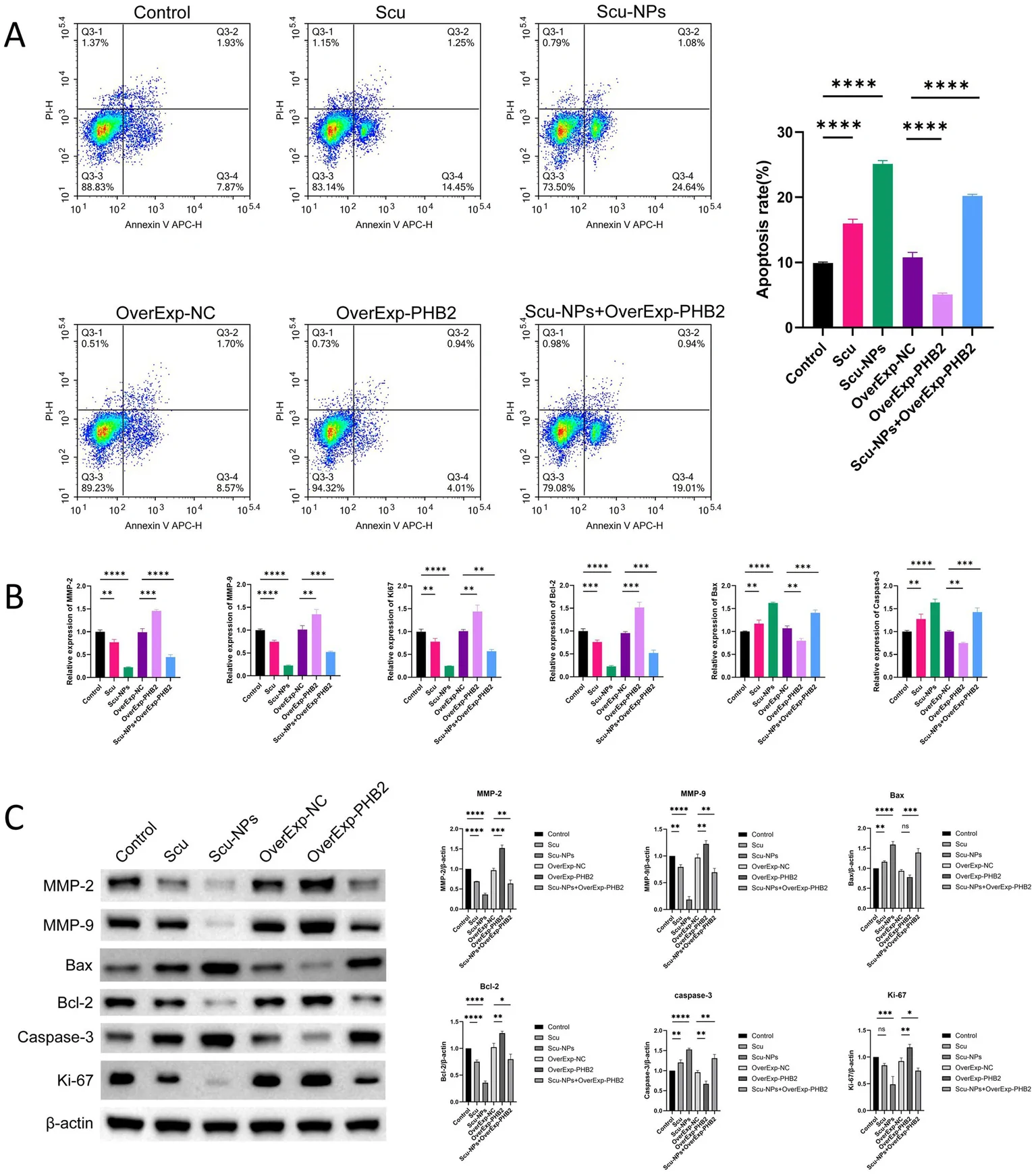
Scu-NPs induce apoptosis and modulate proliferation-, migration-, and apoptosis-related markers in colon cancer cells. **(A)** Flow cytometry analysis of apoptosis using Annexin V-APC/PI staining. **(B)** qPCR analysis of MMP-2, MMP-9, Ki-67, Bax, Bcl-2, and Caspase-3 mRNA levels. **(C)** Western blot analysis of MMP-2, MMP-9, Ki-67, Bax, Bcl-2, and Caspase-3 protein levels. Groups: Control, Scu, Scu-NPs, OverExp-NC, OverExp-PHB2, and Scu-NPs + OverExp-PHB2. Data are presented as mean ± SD. *p < 0.05, **p < 0.01, ***p < 0.001, ****p < 0.0001.

### Scu-NPs suppress colon cancer growth and modulate PHB2-related signaling *in vivo*

In the nude mouse subcutaneous xenograft model, compared with the Control group, tumor growth was mildly suppressed in the Scu group and significantly suppressed in the Scu-NPs group, as reflected by reduced tumor volume and weight. Tumor growth in the Scu-NPs + OverExp-PHB2 group was notably accelerated compared with the Scu-NPs group. HE staining showed densely packed tumor cells with hyperchromatic nuclei and only scattered necrosis in the Control and Scu groups; Scu-NPs induced extensive coagulative necrosis with nuclear pyknosis and karyorrhexis, while the Scu-NPs + OverExp-PHB2 group showed markedly reduced necrosis with better-preserved architecture. Immunohistochemistry revealed strong cytoplasmic staining for PHB2, COX4, and PINK1 in the Control and Scu groups; Scu-NPs decreased PHB2, COX4, PINK1, and Bcl-2 staining while increasing Bax staining, and these changes were partially reversed in the Scu-NPs + OverExp-PHB2 group. Western blot analysis revealed that, compared with the Control group, the Scu-NPs group exhibited significantly downregulated DRP1 and Opa1 protein levels, and upregulated PGC-1α; these changes were attenuated in the Scu-NPs + OverExp-PHB2 group compared with the Scu-NPs group (Figure 6). Collectively, these results indicate that Scu-NPs suppress colon cancer growth *in vivo* through PHB2 downregulation, accompanied by alterations in mitophagy- and apoptosis-related signaling.

**Figure 6:**
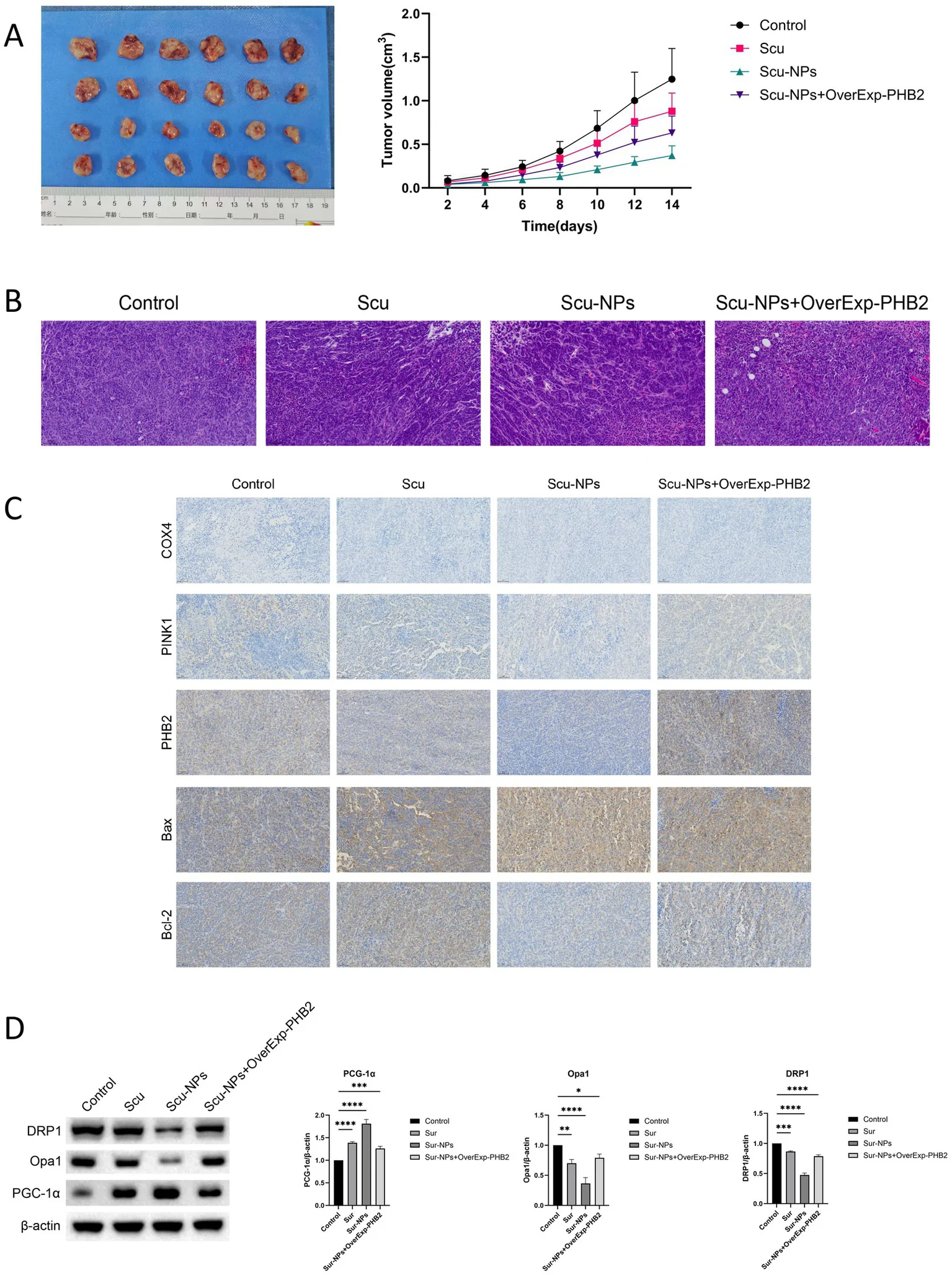
Scu-NPs suppress colon cancer growth and modulate PHB2-related signaling *in vivo*. **(A)** Representative images of xenograft tumors and tumor volume growth curves. *n* = 6 per group. **(B)** HE staining of tumor tissues. **(C)** Immunohistochemical staining for COX4, PINK1, PHB2, Bax, and Bcl-2. **(D)** Western blot and quantitative analysis of DRP1, Opa1, and PGC-1α. Groups: Control, Scu, Scu-NPs, and Scu-NPs + OverExp-PHB2. Data are presented as mean ± SD. *p < 0.05, **p < 0.01, ***p < 0.001, ****p < 0.0001.

### Preliminary exploration of PHB2 in tumor-associated depression and osteotropic migration

To preliminarily explore the potential role of PHB2 in tumor-associated depression and osteotropic migration, we first performed bioinformatics analysis. The results showed that PHB2 is located in the intersection of differentially expressed genes between colon cancer and depression, suggesting it may be a potential molecular interface for cancer–depression comorbidity. In the tumor–depression comorbidity mouse model, compared with the normal group, the model group exhibited significantly increased immobility time in tail suspension and forced swimming tests, and decreased sucrose preference rate. Compared with the model group, the Scu-NPs group showed significantly reduced immobility time and increased sucrose preference rate; these behavioral improvements were partially reversed in mice inoculated with PHB2-overexpressing cells. Furthermore, the osteotropic migration assay showed that, compared with the Control group, migration toward osteoblast-conditioned medium was mildly reduced in the Scu group and more markedly reduced in the Scu-NPs group; PHB2 overexpression increased migration capacity, while Scu-NPs partially reversed this effect (Figure 7). These results preliminarily suggest that PHB2 may be associated with tumor-associated depression and *in vitro* osteotropic migration, and that Scu-NPs may exert potential modulatory effects through PHB2 downregulation; however, these findings require further mechanistic validation.

**Figure 7:**
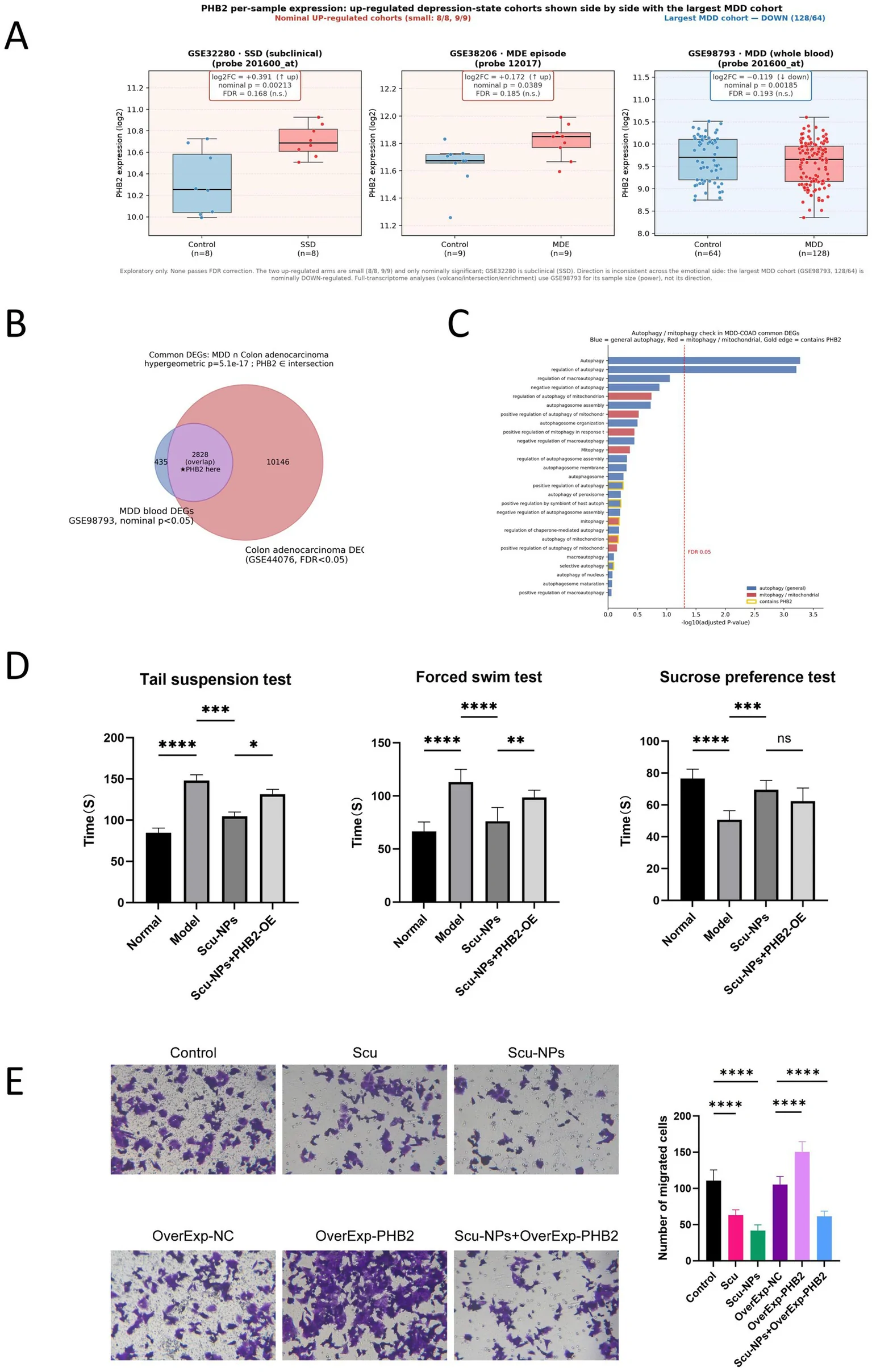
Preliminary exploration of PHB2 in tumor-associated depression and osteotropic migration. **(A)** Bioinformatics analysis indicated an association between PHB2 and emotional disorders. **(B)** Bioinformatics analysis showing the intersection of differentially expressed genes between colon cancer and depression, with PHB2 located in the intersection. **(C)** Enrichr enrichment analysis of the intersection genes revealed that autophagy (KEGG Autophagy, GO regulation of autophagy) was significantly enriched in the intersection. **(D)** Behavioral tests in the tumor–depression comorbidity mouse model, including the tail suspension test, forced swimming test, and sucrose preference test. **(E)** Transwell migration assay of colon cancer cells toward osteoblast-conditioned medium. Groups: **(D)** normal, model, Scu-NPs, and Scu-NPs + PHB2-OE (*n* = 6 per group, randomly assigned by random number table); **(E)** Control, Scu, Scu-NPs, OverExp-NC, OverExp-PHB2, and Scu-NPs + OverExp-PHB2 (three independent experiments). Data are presented as mean ± SD. *P < 0.05, **P < 0.01, ***P < 0.001, ****P < 0.0001. These results are exploratory and require further validation.

## Discussion

Colorectal cancer remains one of the most common malignancies causing cancer-related mortality worldwide (28). Despite continuous advances in surgical intervention, chemotherapy, and targeted therapy, tumor recurrence, metastasis, and therapeutic resistance continue to limit long-term patient benefit, while comorbidities such as depression and bone metastasis further exacerbate the disease burden (29). The present study reveals that integrin αvβ3-targeted scutellarein-loaded nanoparticles (Scu-NPs) suppress colon cancer progression by downregulating PHB2, modulating the PARL–PGAM5–PINK1 axis, inhibiting mitophagy, and inducing mitochondrial dysfunction and apoptosis, providing new mechanistic insights into the regulation of tumor mitochondrial homeostasis by natural product-based nanodelivery systems.

Mitochondrial quality control is a critical mechanism enabling tumor cells to adapt to high metabolic demands (30). Due to rapid proliferation requirements, tumor cells typically experience elevated oxidative stress and mitochondrial damage, and thus rely on protective mechanisms including mitophagy to maintain cellular homeostasis (31, 32). PHB2 plays a dual role in mitochondrial homeostasis: on one hand, it acts as an inner mitochondrial membrane mitophagy receptor, directly mediating clearance of damaged mitochondria through its LIR motif; on the other hand, it preserves the integrity of the PINK1/Parkin signaling pathway by inhibiting the inner membrane protease PARL, thereby protecting PGAM5 and PINK1 from excessive cleavage (33–36). In this study, Scu-NPs significantly reduced PHB2 protein levels, accompanied by elevated PARL, reduced PGAM5/PINK1/Parkin, decreased LC3-II/LC3-I ratio, and P62 accumulation, collectively indicating suppressed mitophagy. Functionally, Scu-NPs induced decreased mitochondrial membrane potential, reduced ATP production, and ROS accumulation, and PHB2 overexpression partially rescued these changes, suggesting that PHB2 is a key target in Scu-NPs-induced mitochondrial dysfunction. Accordingly, we propose that Scu-NPs downregulate PHB2, thereby impairing its direct function as a mitophagy receptor and disrupting PINK1/Parkin signaling through PARL derepression—two convergent mechanisms that may impair mitochondrial clearance and promote the accumulation of dysfunctional mitochondria and subsequent apoptotic signaling. This mitochondrial dysfunction may in turn impair downstream tumor biological behaviors: ATP depletion compromises energy-dependent processes such as cytoskeletal remodeling and cell motility, while excessive ROS accumulation exacerbates mitochondrial damage and activates stress signaling that inhibits proliferation and promotes apoptosis, thereby linking mitophagy inhibition to the suppression of multiple malignant phenotypes (37). Cellular phenotypic and animal experiments further confirmed that Scu-NPs inhibit colon cancer cell proliferation, migration, invasion, and xenograft tumor growth, and PHB2 overexpression partially attenuated these effects.

Beyond its direct antitumor effects, this study preliminarily explored the potential links between PHB2 and tumor-associated depressive-like behaviors as well as osteotropic migration, a cellular phenotype potentially relevant to bone-directed metastatic behavior. Depressive symptoms and bone metastasis represent clinically important complications of colorectal cancer, particularly in patients with advanced disease, and can substantially impair quality of life and prognosis (38–40). On one hand, depression may suppress antitumor immune responses and promote angiogenesis through hyperactivation of the hypothalamic–pituitary–adrenal axis, sympathetic neurotransmitter release, and elevated proinflammatory cytokines, thereby creating a systemic environment favorable for tumor progression and metastasis (41). On the other hand, the persistent bone pain, physical limitation, and psychological distress caused by bone metastasis may in turn induce or aggravate depressive symptoms, suggesting potentially complex bidirectional interactions among tumor, depression, and bone metastasis (40). However, effective multi-target interventions for these comorbidities are currently lacking in clinical practice.

Bioinformatics analysis revealed that PHB2 is located at the intersection of differentially expressed genes between colon cancer and depression. In the tumor-depression comorbidity mouse model, Scu-NPs improved immobility time in tail suspension and forced swimming tests and increased sucrose preference rate, while PHB2 overexpression attenuated these improvements, suggesting that PHB2 may be associated with depressive-like behaviors in the tumor–depression comorbidity model. Although mitochondrial dysfunction is a well-established pathological mechanism in depression, the suppression of mitophagy by Scu-NPs appears paradoxical to behavioral improvement. One possible explanation is that the behavioral improvement may occur indirectly through reduced tumor burden and subsequent attenuation of systemic inflammation, which could in turn influence central nervous system function; however, this hypothesis requires verification through serum inflammatory cytokine profiling and brain tissue analysis. Bone metastasis is a complex, multi-step process involving tumor cell detachment, circulation, homing to the bone niche, and colonization within the bone microenvironment (42). In the osteotropic migration assay, Scu-NPs reduced the migration of colon cancer cells toward osteoblast-conditioned medium, whereas PHB2 overexpression enhanced this migratory capacity and Scu-NPs partially reversed this effect. These findings suggest that PHB2 downregulation may impair the responsiveness of colon cancer cells to osteoblast-derived soluble factors. Whether this effect is mediated by mitochondrial dysfunction and translates into reduced bone homing or colonization *in vivo* remains to be determined. It should be emphasized that the above findings are exploratory in nature; whether the effects of Scu-NPs arise from direct antitumor activity or from comorbidity modulation independent of antitumor effects requires further targeted investigations in neural and bone microenvironments.

Several limitations of this study should be acknowledged. Direct molecular interactions between PHB2 and PARL, PGAM5, and PINK1 require further verification through Co-IP and enzymatic activity assays. Moreover, mitophagy was primarily evaluated based on mitochondrial morphology and mitophagy-related protein expression rather than by dynamic mitophagic flux assays, which should be directly validated in future studies. Extension of the subcutaneous xenograft model to orthotopic or metastasis models would more closely reflect clinical reality. Furthermore, the findings related to depressive-like behaviors and osteotropic migration lack direct molecular evidence from brain and bone tissues and do not distinguish between indirect effects of reduced tumor burden and direct regulation in the central nervous system or bone microenvironment. Additionally, apoptosis was primarily assessed by Annexin V/PI staining and apoptosis-related protein expression, whereas more direct markers such as cleaved Caspase-3 or TUNEL staining were not examined. These issues require future clarification through non-tumor-bearing depression models, tissue-specific PHB2 intervention models, and more direct mechanistic approaches. Nevertheless, the preliminary evidence provided by this study suggests that PHB2 may serve as an important candidate molecular node linking tumor metabolic adaptation, depressive-like behaviors, and *in vitro* osteotropic migration, warranting further investigation.

## Conclusion

In summary, this study demonstrates that integrin αvβ3-targeted β-cyclodextrin-based scutellarein nanoparticles (Scu-NPs), which improve the solubility, stability, and tumor-targeting of scutellarein, suppress colon cancer by downregulating PHB2, modulating the PARL–PGAM5–PINK1 axis, inhibiting mitophagy, and inducing mitochondrial dysfunction and apoptosis. These findings highlight PHB2 as a promising therapeutic target in colon cancer. Moreover, our preliminary exploration suggests that PHB2 may serve as a shared molecular node connecting tumor growth, depressive-like behaviors, and osteotropic migration, providing initial evidence for Scu-NPs as a multi-target strategy addressing both tumor progression and its common comorbidities. Further mechanistic and clinical studies are warranted to validate these findings and evaluate the translational potential of this approach.

## Data Availability

The original contributions presented in the study are included in the article/supplementary material, further inquiries can be directed to the corresponding authors.

## Glossary

αvβ3: Integrin alpha v beta 3
Annexin V-APC/PI: Annexin V-allophycocyanin/propidium iodide
ANOVA: Analysis of variance
ATP: Adenosine triphosphate
Bax: Bcl-2-associated X protein
Bcl-2: B-cell lymphoma 2
Caspase-3: Cysteine-aspartic acid protease 3
CCK-8: Cell Counting Kit-8
CDC4: Cell division cycle 4
COX4: Cytochrome c oxidase subunit 4
CUMS: Chronic unpredictable mild stress
DCFH-DA: 2′,7′-Dichlorodihydrofluorescein diacetate
DMEM: Dulbecco’s modified Eagle’s medium
DRP1: Dynamin-related protein 1
FBS: Fetal bovine serum
GEO: Gene Expression Omnibus
GO: Gene Ontology
GTEx: Genotype-Tissue Expression
HE: Hematoxylin and eosin
IHC: Immunohistochemistry
JC-1: 5,5′,6,6′-Tetrachloro-1,1′,3,3′-tetraethylbenzimidazolylcarbocyanine iodide
KEGG: Kyoto Encyclopedia of Genes and Genomes
Ki-67: Marker of proliferation Ki-67
LC3: Microtubule-associated protein 1 light chain 3
LIR: LC3-interacting region
MACC1: Metastasis-associated in colon cancer 1
MMP-2: Matrix metalloproteinase-2
MMP-9: Matrix metalloproteinase-9
Opa1: Optic atrophy 1
P62: Sequestosome 1 (SQSTM1)
PARL: Presenilin-associated rhomboid-like protein
Parkin: Parkin RBR E3 ubiquitin protein ligase
PGAM5: Phosphoglycerate mutase family member 5
PGC-1α: Peroxisome proliferator-activated receptor gamma coactivator 1-alpha
PHB2: Prohibitin 2
PINK1: PTEN-induced kinase 1
RAGE: Receptor for advanced glycation endproducts
ROS: Reactive oxygen species
Scu: Scutellarein
Scu-NPs: Scutellarein-loaded nanoparticles
SD: Standard deviation
TCGA: The Cancer Genome Atlas
TEM: Transmission electron microscopy
TUNEL: Terminal deoxynucleotidyl transferase dUTP nick end labeling
